# Stereotactic body radiation therapy for hepatocellular carcinoma: From infancy to ongoing maturity

**DOI:** 10.1016/j.jhepr.2022.100498

**Published:** 2022-05-14

**Authors:** Shirley Lewis, Laura Dawson, Aisling Barry, Teodor Stanescu, Issa Mohamad, Ali Hosni

**Affiliations:** 1 Radiation Medicine Program, Princess Margaret Cancer Centre, Toronto, Canada; 2 Department of Radiation Oncology, University of Toronto, Canada; 3 Department of Radiation Oncology, King Hussein Cancer Centre, Jordan

**Keywords:** Hepatocellular carcinoma, HCC, radiation therapy, SBRT, AASLD, American Association for the study of the Liver Diseases, BCLC, Barcelona Clinic of Liver Cancer, DCE, dynamic contrast enhanced, DWI, diffusion weighted imaging, HCC, hepatocellular carcinoma, IVIM, intravoxel incoherent motion, OS, overall survival, PBT, proton beam therapy, PVT, portal vein thrombosis, RFA, radiofrequency ablation, RILD, radiation-induced liver disease, SBRT, stereotactic body radiation therapy, TACE, transarterial chemoembolisation

## Abstract

Hepatocellular carcinoma (HCC) accounts for 90% of liver tumours and is one of the leading causes of mortality. Cirrhosis due to viral hepatitis, alcohol or steatohepatitis is the major risk factor, while liver dysfunction due to cirrhosis is a deciding factor in its treatment. The treatment modalities for HCC include liver transplant, hepatectomy, radiofrequency ablation, transarterial chemoembolisation, transarterial radioembolisation, targeted therapy, immunotherapy, and radiation therapy. The role of radiation therapy has been refined with the increasing use of stereotactic body radiation therapy (SBRT). Trials over the past two decades have shown the efficacy and safety of SBRT in recurrent and definitive HCC, leading to its acceptance and adoption in some more recent guidelines. However, high quality level I evidence supporting its use is currently lacking. Smaller randomised trials of external beam radiation therapy suggest high efficacy of radiation therapy compared to other treatments for patients with unresectable HCC, and phase III trials comparing SBRT with other modalities are ongoing. In this review, we discuss the rationale for SBRT in HCC and present evidence on its efficacy, associated toxicity, and technological advances.

**Key points**

• SBRT is an emerging treatment modality offering potentially curative local therapy for HCC.
• SBRT is applicable across BCLC stages (bridge to transplant, BCLC A, BCLC B, portal vein thrombosis) as an alternative treatment strategy to TACE/RFA, or in recurrent tumours as salvage therapy.
• The recent prospective and retrospective studies have shown the safety and efficacy of SBRT with 2-year local control ranging from 68-95%.
• Smaller randomised trials of external beam radiation therapy suggest high efficacy of radiation therapy compared to other treatments for patients with unresectable HCC, and phase III trials comparing SBRT with other modalities are ongoing.

## Introduction

Hepatocellular carcinoma (HCC) is the most common malignant liver tumour.^1^ As per GLOBOCAN 2020, HCC is the 6th most common cancer worldwide and the third leading cause of cancer-related mortality.^2^ Cirrhosis is the primary underlying aetiology and is commonly caused by viral hepatitis (hepatitis B and C), alcohol and non-alcoholic fatty liver disease secondary to obesity or diabetes mellitus.^3^ The global burden of HCC increased by 75% from 1990 to 2015, and it is expected that the annual increase by 2030 will be 35% greater than that in 2005.^3^^,^^4^ There is a wide variation in incidence and risk factors among countries, with the highest growth expected in North America.^4^ Age at presentation varies, with younger patients affected in endemic areas, and there is a male preponderance. The Barcelona Clinic Liver Cancer (BCLC) staging system is the most commonly adopted staging tool for HCC; it incorporates liver function with performance status and guides treatment strategy, and it is endorsed by the American Association for the Study of Liver Diseases (AASLD) and the European Association for the Study of the Liver (EASL).^1^^,^^5^^,^^6^

The treatment of HCC is challenging and requires a multidisciplinary approach to decision making.^7^ Despite adopting screening strategies for at-risk populations, over 50% of new cases present at an advanced stage.^8^ Various treatment modalities are available, such as liver transplant, hepatectomy, radiofrequency ablation (RFA), microwave ablation, percutaneous ethanol injection, transarterial chemoembolisation (TACE), transarterial radioembolisation, radiation therapy, targeted therapy and immunotherapy.^6^ Surgery and liver transplant are considered primary curative options; however, only about 20% of cases are eligible for such therapies due to a shortage of liver donors, long wait times to transplant, underlying liver dysfunction and/or advanced stage at the time of presentation.^9^ Unlike liver transplantation, resection does not treat the underlying cirrhosis present in the liver. Tumour recurrence is also more frequent after resection, with development of new lesions requiring further salvage treatments in the limited stage. About 20-25% of patients are not fit for any active treatment due to end-stage liver disease, comorbidities, age and advanced stage cancer.^10^ The 5-year survival for HCC overall is 10-12%, while rates of 70-75% are reported in those who undergo liver transplant, with some even surviving to 10 years.^11^^,^^12^ Screening in high-risk populations is the key to improving survival.^13^^,^^14^ SBRT is an emerging treatment modality offering a potentially curative local therapy for HCC. This review article will address its role in HCC, presenting evidence on its efficacy and toxicity, and looking at technological advances.

## Radiation for HCC: historical background and mechanism of action

Historically, external beam radiotherapy was used to treat HCC, although it was mainly applied as whole liver treatment in the context of palliative care.^15^ It has not been a well-accepted local liver-directed therapy compared to RFA or TACE, due to concerns around radiation-induced liver disease (RILD).^16^ While RILD was a risk with whole liver treatment, new techniques that target radiation within the liver have allowed for a reduction in mean doses to less than 28 Gy (in conventional fractionation, *i.e.* 2 Gy per fraction), which were associated with just a 5% risk of RILD.^17^ Techniques like 3D conformal radiotherapy and intensity-modulated radiation therapy are better at sparing the non-tumorous liver parenchyma than conventional 2D techniques. The most used doses for 3D conformal radiotherapy and intensity-modulated radiation therapy techniques ranged from 45 Gy to 66 Gy in 10-33 fractions at 1.8-2.5 Gy per fraction, with studies reporting 2-year survival rates of 40% to 70% and that a higher dose to the tumour was associated with superior response and survival.[18], [19], [20], [21], [22]

Further refinement in radiation technology, availability of motion management strategies and improvement in imaging for localisation and delivery have resulted in the transition to SBRT. Since its inception and first application in liver tumours by Blomgren *et al.*, the use of SBRT has increased over the years.^23^ SBRT refers to the delivery of high doses of highly conformal radiation to the tumour in 1-5 fractions. The advantage is the ability to target the tumour precisely with a steep dose gradient limiting the dose to the non-involved liver and surrounding normal structures. This enables the delivery of higher biologically effective doses than conventional fractionation.

Radiation acts through direct and indirect mechanisms resulting in double-stranded breaks in the DNA. The radiation dose of 10 Gy in a single fraction or 20-60 Gy in multiple fractions causes vascular injury with resultant tumour hypoperfusion, hypoxia and indirect cell death.^24^ A higher dose per fraction causes damage to the vascular endothelium, with consequent apoptosis and vascular leakage.^25^ There is also potential to injure the radioresistant stem cell in the perivascular niche. Radiation also causes immunostimulatory effects resulting in immunogenic cell death. Furthermore, radiation releases a pool of tumour-associated antigens which activate dendritic cells, which in turn, activate and prime CD8 T cells that mount an antitumour response and enhance immune infiltration into the tumour microenvironment.^26^

## Evidence and indications for SBRT in HCC

SBRT has been used successfully as an alternative to TACE/RFA, or as salvage therapy in recurrent tumours.[27], [28], [29] The patients included in the studies were often heavily pre-treated and had large lesions, comorbidities, and poor liver function that made RFA/TACE unsuitable. This inherent bias has led to a lack of observed survival benefit compared to other modalities. The recent prospective and retrospective studies have shown comparable outcomes with local control at 2 years ranging from 68-95%, as shown in Table 1, which summarises the salient trials of SBRT in HCC.^27^^,^[30], [31], [32], [33], [34], [35], [36], [37], [38], [39], [40], [41], [42], [43], [44] Recent phase III trials with proton beam therapy and external beam radiotherapy have demonstrated safety and efficacy.^30^^,^^45^^,^^46^ Due to the lack of completed randomised trials (and hence level 1 evidence) demonstrating the efficacy of SBRT, it currently fails to find a place in liver treatment guidelines.^16^^,^^47^^,^^48^ However, SBRT is applicable in HCC with recently published American Society of Radiation Oncology (ASTRO) guidelines formalising potential treatment options.^49^

**Table 1:** **Select prospective and retrospective series showing outcome with stereotactic body radiotherapy**.

Study	Patient number	Quality/type of study	Indication/stage (BCLC)	Dose and fractionation	Follow up	Outcomes (LC/OS)	Toxicity (Grade 3 liver/GI)	Study conclusion	Level of Evidence∗
Kim *et al*., 2021^30^	72	Phase III randomised trial- Proton vs RFA	0-C	66Gy/10Fr (Protons)	51.6m	2y LC: 92.8% 2y OS: 91.7%	none	Proton beam therapy was non-inferior to RFA and was tolerable.	II
Yoon *et al*., 2020^31^	50	Prospective Phase II trial	0 and A (small HCC)	45 Gy/3#	47.8 m	5y LC: 97.1% 5y OS: 77.6%	4%	SBRT showed good results for ablation of small HCC with minimal toxicity.	IV
Labrunie *et al*., 2020^32^	43	Prospective Phase II trial	A-C	45 Gy/3#	4 y	2y LC: 94% 2y OS: 69%	5%	LC and OS was promising in HCC treated with SBRT.	IV
Jang *et al*., 2020^33^	65	Prospective Phase II trial	0-C	60 Gy/3#	41m	2y LC:97% 2y OS: 84%	2%	SBRT for HCC was well tolerated.	IV
Park *et al*., 2020^34^	290	Prospective Phase II trial	0-A	30-60Gy/3#	38.2m	5y LC: 91.3% 5y OS: 44.9%	8.8%	SBRT is an ablative option for small HCC.	IV
Mathew *et al*., 2020^35^	297	Retrospective	0-D	27-60Gy/3-6#	19.9m	3y LC: 87% 3y OS: 39%	16%	SBRT provides good LC and OS in HCC when it is unsuitable or refrac-tory to other locoregional treatment.	VI
Kim *et al*., 2019^36^	32	Prospective Phase I/II trial	A-B	36-60 Gy/4#	27m	2y LC: 87% 2y OS: 81.3%	None	SBRT is a safe treatment for HCC.	IV
Hara *et al*., 2019^37^	143	Retrospective	0-C	35-40Gy/5#	30.2m	3y LC: 95.6% 3y OS: 63.6%	8.2%	SBRT for HCC provides results comparable to RFA and acceptable alternative to RFA.	VI
Feng *et al*., 2018 ^38^	90	Prospective Phase II trial	A-C	23-60Gy/5#	37m	2y LC:95%	7%	Individualized adaptive RT was safe and provided good LC.	IV
Takeda *et al*., 2016^39^	90	Prospective Phase II trial	0-C	35-40 Gy/5#	41.7m	3y LC: 96.3% 3y OS: 66.7%	6%	SBRT achieved high LC with accepatable toxicity in solitary HCC.	IV
Lasley *et al*., 2015^40^	59	Prospective Phase I/II trial	A-C	48 Gy/3# in CPA 40 Gy/5# in CPB	33.3m	3y LC: 82-91% 3y OS: 26.1- 61.3%	20%	SBRT is a safe treat-ment for HCC.	IV
Scorsetti *et al*., 2015^41^	43	Prospective observational	A-C	48-75 Gy/3# to 36 Gy/6#	8m	2y LC: 64% 2y OS: 45%	16%	SBRT is safe with acceptable LC and toxicity.	IV
Bujold *et al*., 2013^42^	102	Prospective Phase I and II trials	A-C	24-56 Gy/6#	31.4m	1y LC: 87% Med OS: 17m	30%	Results provide strong rationale for studying SBRT as local treatment option in randomised phase III trials.	IV
Kang *et al*., 2012^27^	47	Prospective Phase II trial	A-C	42-60 Gy/3#	17m	2y LC: 94.6% 2y OS: 68.7%	6.4%	SBRT after incomplete TACE provides promising response and LC.	IV
Andolino *et al*., 2011^43^	60	Prospective observational	A-C	40-44 Gy/3-5#	27m	2y LC: 90% 2y OS: 67%	20%	SBRT is safe non-invasive option for HCC <6cm.	IV

GI, gastrointestinal; LC, local control; OS, overall survival.

∗ ^44^

### SBRT in neoadjuvant setting – bridge to transplant

The standard curative option in early-stage HCC is liver transplantation. While only a few patients are eligible for transplant, many will have a long wait for a donor liver and run the risk of dropping off the list because of disease progression. AASLD recommends bridging therapy when waiting time is ≥6 months, and patients are often considered for the same when listed.^50^ The aim of local therapy in this setting is to prevent progression and downsize the tumour to maintain the eligibility for transplant. RFA, microwave ablation, percutaneous ethanol injection, or TACE are commonlyly used. A meta-analysis by Kulik *et al.* showed that bridging therapy before liver transplant led to reduced dropout rates and improved survival outcomes.^51^ The application of SBRT as bridging therapy is relatively new, with only a few institutional series reporting on its safety and efficacy.^52^^,^^53^ One of the earliest reports, from the University of Toronto, demonstrated the safety of conformal radiation therapy (8.5-33 Gy in 1-6 fractions) as bridging therapy, with 5 of 10 patients undergoing transplant after radiation without complications.^54^ Connor *et al.* treated 10 patients with SBRT (median 51 Gy in 3 fractions) before transplant, and 27% had a complete response, while the remaining 73% had a partial response or stable disease.^55^ The median time to transplant was 113 days with no increase in postoperative morbidity. The overall survival (OS) and disease-free survival were 100% at 5 years.

Few studies compared radiation with TACE or RFA as a bridging therapy. Mohammed *et al.* compared the pathological complete response rates (pCR) among the bridging treatments (SBRT, RFA, TACE and transarterial radioembolisation) and showed lower pathological complete response rates with SBRT than other modalities (28.5% *vs.* 40-75%).^56^ Survival rates were similar. A phase II randomised trial by Nugent *et al.* showed lesser rates of toxicity and retreatment at 1 year with SBRT compared to TACE (0 *vs.* 38.9%).^57^^,^^58^ The randomised trial by Bush *et al.* compared TACE with proton beam therapy (PBT) in 69 patients with HCC that met the Milan or San Francisco criteria for transplant. The interim analysis showed similar OS and complete response rates with a trend to improved local control and progression-free survival with PBT.^59^ Sapisochin *et al.* evaluated the outcomes of 379 patients following transplant and examined the efficacy of SBRT *vs.* TACE or RFA.^60^ The patients with SBRT received relatively lower prescription doses (36 Gy/6 fractions) and a higher proportion had poor liver function. The dropout rates and complications were similar among the groups. Though recurrence rates were higher with TACE and SBRT than with RFA, the 1-, 3- and 5-year OS rates were identical among the groups (83%, 75% and 75% with SBRT *vs*. 96%, 75% and 69% with TACE, and 95%, 81% and 73% with RFA group, *p* = 0.7). SBRT appears to be a good alternative to other bridging therapies, particularly for those with poor liver function. The results of 2 phase II trials comparing SBRT with TACE are awaited (NCT02470533, NCT02182687), and a phase III trial is ongoing (NCT03960008).

### SBRT in the definitive setting

#### Early-stage HCC (BCLC 0/A)

RFA is the recommended first-line treatment for HCC less than 3 cm, if unresectable or not suitable for transplant, with 3-year local control rates of over 90%.^61^ The application of RFA is challenging in situations where the tumour is near vessels (heat sink effect) or the hilum or dome of the diaphragm (risk of complications), or if the tumour is large (resulting in incomplete ablation [2-60%] and poor outcomes).^62^ SBRT provides reasonable local control and survival rates (3-year local control: 68-97% and 3-year survival: 39-84%) when RFA is contraindicated or in a recurrent setting post-RFA or TACE.^63^ A large retrospective North American study by Matthew *et al.* reported outcomes of 297 high-risk patients with HCC treated with SBRT from 2003 to 2016; patients were either not candidates for RFA/TACE or had recurrent/residual disease without vascular invasion after RFA/TACE(35). The 3-year OS rate was 39% with a 13% recurrence rate despite large tumours. The toxicity was acceptable with Child-Pugh progression by 2 points at 3 months noted in 16% with no RILD. Even in treatment-naïve small HCC (1-3 cm), SBRT has shown promising outcomes in recent studies from Korea. Park *et al.* retrospectively analysed the long-term outcomes associated with SBRT for small HCC and reported 5-year local control and OS rates of 91% and 45%, respectively, at a median follow-up of 38.2 months.^34^ Higher local control (93%) was seen in tumours <3 cm and grade 3 liver toxicity was observed in only 2.2% of patients.

Of the studies that compared SBRT with RFA, some showed equivalent results, and others showed superiority of one modality over another.^37^^,^[64], [65], [66] Bias in patient selection and lack of liver function characteristics are limitations of these observational studies. A phase III randomised non-inferiority trial by Kim *et al.* compared PBT with RFA in recurrent HCC (n = 144) and found the 2-year local progression-free survival with PBT was non-inferior to RFA (92.8% for PBT *vs.* 83.2% for RFA).^30^ The 4-year survival was similar between the 2 arms. Su *et al.* showed superior local control and progression-free survival with SBRT (n = 167) compared to TACE (n = 159) in 326 patients with inoperable BCLC-A stage HCC.^67^ No phase III randomised trials compared SBRT with RFA, TACE or surgery for early-stage primary HCC. Three recent meta-analyses have aimed to provide objectivity to inform decision making.[68], [69], [70] The meta-analysis by Pan *et al.* included 10 studies comparing SBRT with RFA in patients with treatment-naïve HCC and showed superior 1- and 3-year local control with SBRT.^68^ The 2-year OS was possibly lower with SBRT due to variation in baseline liver function and tumour size. After eliminating reporting bias, the secondary analysis showed equivalent 2-, 3- and 5-year OS rates between the 2 modalities. These conclusions of higher local control and comparable survival with SBRT were supported by Wang *et al.*, while Lee *et al.* reported similar control but a survival benefit with RFA.^69^^,^^70^ Two ongoing randomised trials are comparing SBRT with RFA in small HCC in a definitive and recurrent setting (NCT03898921, NCT04047173).

#### Intermediate and advanced stage HCC (BCLC B/C)

In an unresectable (large tumour size, multifocal, portal vein thrombosis and oligometastatic) patient cohort, SBRT is feasible. Several retrospective and prospective series showed acceptable local control (2-year: 65-95%) and OS (2-year: 40-80%) rates with SBRT (Table 1). The studies vary widely with respect to SBRT doses used, baseline liver function, and portal vein thrombosis (30-65%). The dose used depends on the location and size of the tumour, baseline liver function and the dose constraints achieved while planning. Patient selection is crucial as those with baseline Child-Pugh class B8 and above are at greater risk of toxicity, as such SBRT is generally unsuitable in these patients.^71^ While most use 5-6 fraction schedules, recent trials have used 3 fraction schedules with reasonable local control rates.^32^^,^^33^ It is unclear whether a higher dose affects outcomes.^72^^,^^73^

TACE is a preferred treatment modality for patients with BCLC B HCC, and prospective trials have demonstrated its efficacy, leading to its incorporation into the BCLC treatment paradigm.^74^ Few studies have compared TACE with SBRT. Sapir *et al.* reported outcomes of a propensity score analysis of 209 patients with 1-2 tumours who underwent TACE (n = 84) or SBRT (n = 125).^75^ The 2-year local control rate was superior with SBRT compared to TACE (91% *vs.* 23%, *p* <0.001), with similar survival rates (2-year OS 34.9% *vs*. 54.9 %, *p =* 0.21). Similarly, a propensity score analysis by Bettinger *et al.*, comparing TACE with SBRT in HCC BCLC B/C, showed comparable 1-year local control (82.9% *vs.* 84.8%, *p =* 0.8) and 1 year OS (52.9% *vs.* 53.1%) rates.^76^ These studies suggest SBRT is an alternative approach to TACE in patients with BCLC B HCC. Ongoing studies are comparing TACE with SBRT (NCT02470533, NCT03338647). The addition of SBRT to TACE has also been explored in various studies.^77^ A meta-analysis by Zhao *et al.* suggests higher response, local control, and survival rates with TACE and SBRT *vs.* SBRT alone.^78^ Randomised studies comparing TACE with TACE and SBRT in unresectable HCC are ongoing (NCT03895359 and NCT02794337).

While systemic therapy is standard of care for portal vein thrombosis (PVT), radiation therapy appears to provide sustained local control in a substantial proportion of patients. A randomised trial by Yoon *et al.* compared the combination of TACE and radiation with sorafenib in 90 patients with Child-Pugh A HCC with PVT and showed improved progression-free survival (86.7% *vs.* 34.3%; *p <*0.001), time to progression (31.0 *vs.* 11.7 weeks; *p <*0.001) and OS (55.0 *vs.* 43.0 weeks; *p* = 0.04) with TACE-RT.^45^ Munoz-Schuffenegger reported the long-term outcomes of 128 patients with HCC and PVT treated with SBRT in a single institution from 2003 to 2016.^79^ With a dose of 27-54 Gy in 5 fractions, 1-year local control was 87.4% and median OS was 18.3 months. The RTOG 1112 is a phase III trial comparing SBRT with sequential sorafenib *vs.* sorafenib alone, and the results are awaited (NCT01730937). A retrospective study by Bettinger *et al.* compared SBRT with sorafenib in advanced HCC (recurrent, metastatic, and advanced) in a propensity score analysis.^80^ SBRT showed improved median overall survival compared to sorafenib (17 *vs*. 9.6 months).

### SBRT/RT in the palliative setting

Patients with diffuse HCC or those who are ineligible for focal treatment due to poor function or diffuse distant metastases are usually symptomatic and present with pain. The studies of whole liver radiation therapy indicate palliation with 20-30 Gy in 45-80% of cases.^81^^,^^82^ In a phase II trial by Soliman *et al.*, 21 patients with HCC were treated with 8 Gy in a single fraction to the whole liver or tumour.^83^ At 1 month, 48% had symptom improvement with quality-of-life improvements in 21-29%. A phase III randomised study comparing palliative radiotherapy with best supportive care is ongoing (NCT02511522).

## Toxicity following SBRT

Liver SBRT is challenging due to the risk of damage to the surrounding uninvolved liver and neighbouring organs like bile ducts and gastrointestinal organs (*i.e.*, oesophagus, stomach, duodenum, and bowel). Maintaining the balance between the risk of toxicity and tumour control is the key. Table 2 shows commonly used constraints for SBRT planning. In cases of close abutment with these organs, the dose to the tumour is often compromised to satisfy the dose constraints and limit toxicity.^49^

**Table 2:** **Dose constraints for stereotactic body radiation therapy planning**.

Organ at risk	Constraint for 3 fractions	Constraint for 5 fractions
Uninvolved liver (non-cirrhotic)^137^		
Mean dose	<12-15 Gy	<15-18 Gy
Dose to ≥700 cm^3^	<19 Gy	<21 Gy
Uninvolved liver (Child-Pugh class A)^40^^,^^85^^,^^138^		
Mean dose	<10-12 Gy	<13-15 Gy
Dose to ≥700 cm^3^	—	<15 Gy
Uninvolved liver (Child-Pugh class B)^40^^,^^85^^,^^138^		
Mean dose	None	<8-10 Gy
Dose to ≥500 cm^3^		<10 Gy
Stomach^137^		
D 0.03 cm^3^	<22 Gy	<32 Gy
D 10 cm^3^	<16.5 Gy	<18 Gy
Duodenum^137^^,^^139^		
D 0.03 cm^3^	<22 Gy	<32 Gy
D 5 cm^3^	<16.5 Gy	<18 Gy
Small bowel^137^^,^^139^		
D 0.03 cm^3^	<22 Gy	<32 Gy
D 5 cm^3^	<18 Gy	<19.5 Gy
Large bowel^137^^,^^139^		
D 0.03 cm^3^	<28 Gy	<34 Gy
D 20 cm^3^	<24 Gy	<25 Gy
Common bile duct^88^		
D 0.5 cm^3^	40 Gy	40 Gy

### Liver toxicity

RILD is the most dreaded toxicity of SBRT. It includes classic and non-classic RILD.^84^ The classic RILD is a triad of anicteric hepatomegaly, ascites, and elevated liver enzymes and alkaline phosphatase (2 times the normal) occurring 2 weeks to 3 months after radiation. The pathological hallmark is venoocclusive disease. The non-classic RILD occurs in existing liver disease and manifests as jaundice and raised transaminases (5x the upper limit of normal). In the modern HCC series, the incidence of classic RILD is less than 5%. The most common reported form of liver toxicity is non-classic RILD with change in Child-Pugh score by ≥2 points, which occurs in 10-30% of patients 1-3 months after SBRT. Velec *et al.* report toxicity outcomes in 101 patients treated with 6-fraction SBRT in clinical trials at the Princess Margaret Cancer Centre, with liver toxicity (increase in Child-Pugh by 2 points) occurring in 26% of cases.^85^ No patients developed classic RILD. Baseline liver function and higher liver dose (mean dose >16.9 Gy and dose to 800 cm^3^ of liver >14.3 Gy) were predictive of worsening of Child-Pugh score 3 months post SBRT. In patients with Child-Pugh B or C treated with SBRT, Child-Pugh score declined by ≥2 points at 3 months in over 60%.^71^

### Luminal gastrointestinal structure toxicity

**Fig. 1:**
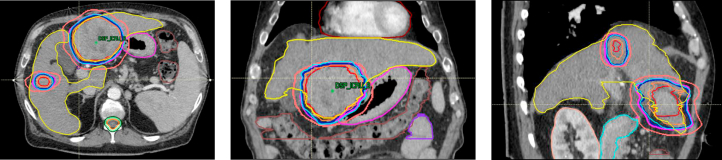
Planning CT in arterial phase (axial, coronal, and sagittal cuts) showing SBRT plan for HCC – 3 lesions treated with a dose of 27.5 Gy in 5 fractions. 60-year-old gentleman diagnosed with multifocal HCC, BCLC B with Child-Pugh B8 with 3 lesions treated with SBRT as a bridge to transplant. Red- Gross tumour volume, Cyan- Planning target volume, Blue: 100% isodose line, Pink: 95% isodose line and Orange:70% isodose line.

The luminal gastrointestinal structures are vulnerable to injury because of their proximity to liver tumours and changes linked to portal hypertension-related gastroduodenopathy. This common toxicity manifests as ulcers, fistulas or bleeding, and the rate of grade 3 toxicity was reported to be 5-10%.^27^^,^^32^^,^^86^ Selection of tumours >1 cm away from the gastrointestinal structures is recommended. Often the dose to the tumour may have to be compromised to meet the organs-at-risk constraints (Fig. 1).

### Biliary tract toxicity

The common forms of central hepatobiliary toxicity (HBT) are biliary stricture, biliary obstruction, hepatobiliary infection, or sepsis. The structures in the central hilum of the liver, such as the hepatobiliary tract and portal vein, behave as serial structures. Toesca *et al.* reported grade 3 HBT in 17.5% of patients with HCC, while none had strictures.^87^ HBT was highly correlated with the dose to the central structures. The volumes receiving 40 Gy (>37 cm^3^) and 30 Gy (>45 cm^3^) were predictors of grade 3 HBT.^87^ Eriguchi *et al.* suggest that 40 Gy in 5 fractions is safe for the biliary tract, with only 2 of the 50 treated patients having asymptomatic biliary stenosis (both treated at a dose >40 Gy).^88^

### Chest wall toxicity

Chest wall toxicity manifests as rib pain and rib fractures associated with peripherally located HCC. Chest wall pain has been reported in up to 21% and rib fracture in about 7-8% of patients.^89^ Chest wall toxicity is commonly self-limiting with analgesics. The high dose (Dmax <50 Gy and 40 Gy <5 cm^3^) should be limited when treating close to the chest wall.^89^

## Technical advances in SBRT for HCC

Dose escalation to the tumour while minimising dose to the organs at risk is the primary aim of radiation therapy. Over the last 2 decades, the leading technological advances in radiation oncology aim to maximise the therapeutic ratio by driving this fundamental aim, namely magnetic resonance-guided radiotherapy and PBT.

### MRI-guided radiotherapy

MRI-guided radiotherapy is a novel technology that aims to conform and adapt treatment to the tumour by taking into account the intra- and inter-fraction variations in the spatial and functional characteristics of the target and organs at risk and enabling dose escalation.^90^ With improved localisation and management of organ motion, there is potential for planning target volume margin reduction resulting in smaller irradiated volumes. The advantages of MRI-guided radiotherapy are superior soft-tissue visualisation, daily online adaptive replanning and real-time monitoring of tumour motion with no additional imaging dose. Few studies have reported on the application of MRI-guided liver SBRT.[91], [92], [93], [94] Rosenberg *et al.* presented the results of multi-institutional experience with MRI-guided liver SBRT in 26 patients, including 6 patients with HCC.^91^ At a median follow-up of 21.2 months, freedom from local progression was 100% in patients with HCC with a median dose of 50 Gy in 5 fractions. One patient, with a large tumour (>900 cm^3^) and high mean liver dose (18.9 Gy), developed portal hypertension with Child-Pugh worsening from A to C. Feldman *et al.* showed the feasibility of respiratory-gated delivery of MRI-guided liver SBRT in 26 patients with HCC.^92^ Treatment was well tolerated with stable to partial response in all except 1 patient. A phase I trial by Henke *et al.* showed the safety of stereotactic MRI-guided online-adaptive radiotherapy (SMART) in abdominal malignancies (HCC, n = 4).^95^ It allowed for improved planning of target volume coverage with organ sparing based on the anatomy of the day, and dose escalation was feasible in 10 patients. While this technology is promising, long-term follow-up showing improvement in late toxicity is needed. Future trials should explore dose escalation in HCC and compare it with conventional techniques.

### Proton beam therapy

PBT is attractive for use in HCC due to the dosimetric advantage of the absence of an exit dose, resulting in better organ sparing and the potential for dose escalation.^96^ This is likely beneficial, particularly for HCC close to gastrointestinal organs and in patients with borderline liver function. The most significant evidence on PBT is reported from the University of Tsukuba, Japan.^97^ They used 3 different PBT dose fractionation protocols based on proximity to gastrointestinal organs and porta hepatis. They reported a 5-year local control rate of 80% and a 5-year OS rate of 48% in 266 patients treated from 2001 to 2007. Several prospective phase II trials the efficacy of protons in HCC, with recent series showing 2-year local control in around 95% of patients.[98], [99], [100], [101] In a retrospective comparison of photons with protons, protons were associated with improved survival (median OS 31 *vs.* 14 months) and a lower risk of post-treatment liver decompensation.^102^ A phase III randomised trial comparing photons with protons is ongoing (NCT03186898). Protons have shown impressive results in comparison with other modalities as well. A randomised trial comparing PBT with TACE showed a trend to improved local control with PBT, with similar survival rates.^59^ A recent phase III randomised trial by Kim *et al.* showed 2-year local progression-free rates for PBT were non-inferior to RFA (92.8% *vs.* 83.2%).^30^ Most of these trials used the passive beam scattering method and hypofractionated protocols (10-20 fractions). Recent studies explore the pencil beam scanning method with intensity-modulated proton therapy and shorter stereotactic fractionation (5 fractions).^103^

## Future directions

SBRT dose and fractionation for HCC varies based on tumour size, liver function and institutional guidelines. Dose escalation is an area of active investigation and higher local control is suggested with biologically effective doses >100 Gy in HCC.^73^ It is unclear whether there exists a clear dose response for local control, with differing opinions in the literature.^72^^,^^86^^,^^104^ Single fraction SBRT (26-40 Gy) led to good local control in phase I/II trials for liver metastases, while reports on its application in HCC are limited.[105], [106], [107] The critical luminal structures and healthy non-tumorous liver often limit the dose delivered. MRI-guided SBRT is well suited for dose escalation as it offers high accuracy with superior soft tissue contrast for tumour delineation, adaptive online replanning with improved organ sparing and potential for reduction of treatment margins. MRI-based SBRT with respiratory gating or breath hold technique, with visualisation of the target during delivery, maximises sparing of healthy liver and reduces inter and intrafraction uncertainties.^93^ Future trials should explore high biologically effective doses (>100 Gy) of SBRT in 1-5 fractions, utilising MRI-guided SBRT, and assess their impact on OS.

Current planning in radiotherapy including MRI-based radiotherapy involves a mixed CT-MRI workflow, where CT provides the electron density information required for dose calculations. An MRI-only planning approach has been described where a synthetic CT was generated from MRI data to facilitate radiation therapy planning and obviate the need for an additional CT scanning session. The main advantages of this approach are efficiency, cost effectiveness, avoidance of radiation exposure and geometric uncertainties associated with CT-MR co-registration.^108^ Reports in prostate cancer suggest that MRI-only workflows are safe and feasible and result in a reduction of systematic error by 2-3 mm.^109^^,^^110^ Research on MRI-only workflows in SBRT for HCC or abdominal targets is in its infancy.^111^^,^^112^ The reduction in margins provided by MRI-based workflows coupled with online adaptive replanning of MRI-based SBRT makes dose escalation feasible with maximal organ sparing and high accuracy – hence, this approach should be investigated in future prospective trials.

HCC is associated with high intratumoural and interpatient heterogeneity, which contributes to metastases, recurrence and resistance.^113^ *TP53, CTNNB1, and TERT* mutations are key driver mutations seen in HCC and key molecular subclasses have been proposed based on transcriptome analysis.^114^ *In vitro* studies suggest high p53 expression to be a marker for response to radiation.^115^ Liu *et al.* showed superior survival in patients with HCC treated with TACE+RT compared to TACE alone and this was correlated with higher p53 downregulation with TACE+RT than TACE alone (*p* <0.05).^116^ Enhanced wnt/β-catenin pathway activation and cancer stem cells are associated with radio resistance in HCC.^117^ Huang *et al.* demonstrated the radiosensitising effect of the wnt/β-catenin inhibitor ICG-001 in nude mouse tumour models.^118^ Biomarkers for liver injury/toxicity have been studied. Ng *et al.* found that proinflammatory soluble cytokine receptors (higher levels of soluble TNFRII and lower levels of soluble CD40L) early during SBRT were correlated with liver toxicity and predicted increased risk of death following SBRT.^119^ Clinical studies exploring molecular markers of response and toxicity will enable personalisation of SBRT.

MRI functional imaging sequences like diffusion weighted imaging (DWI) and more recently, intravoxel incoherent motion (IVIM) provide additional information on cellular density and perfusion in the tumour microenvironment.^120^ IVIM is an emerging field and potentially superior to traditional DWI as it can be used to obtain information related to microcirculatory perfusion in tumour tissue. Dynamic contrast enhanced (DCE) MRI is used to measure changes in tumour vascular permeability.^121^ IVIM and DWI parameters have been used to predict the histological differentiation of HCC and evaluate the expression of enzymes or factors in the tumour microenvironment.^122^^,^^123^ IVIM along with DCE MRI biomarkers (before and early after treatment) are predictive of response to treatment and survival in HCC.[124], [125], [126] Future research should investigate the incorporation of IVIM and DCE into SBRT planning for HCC and use the parameters to identify and tailor the high dose to at-risk regions, leading to personalised dose escalation and improvement in the therapeutic ratio.

Radiomics involves deeper analysis of imaging data and uses quantitative data features and texture analysis to provide insights into tumour heterogeneity.^127^ It is a promising field that could be integrated into radiation planning, monitoring of tumour response and prediction of toxicity. Radiomic features have been explored as potential biomarkers to predict prognosis in HCC and are an emerging field that could enable treatment personalisation.^128^^,^^129^ Trials are underway on the use of radiomics to assess prognosis and to guide surgical decisions (NCT02757846, NCT03917017). Incorporation of radiomic features in target delineation may improve accuracy by identifying high-risk areas of microscopic spread beyond the gross tumour, predicting microinvasion and even enabling auto segmentation.^130^ With MRI-based radiotherapy, application of radiomics to imaging data for planning may help to improve our understanding of tumour heterogeneity and allow for the early assessment of response.^131^ In the era of precision medicine and technological advances, future trials should try to personalise SBRT based on individual genomic or radiomic signatures.

Immunotherapy is the recommended first-line treatment for locally advanced unresectable HCC.^132^ SBRT can induce an antitumor immune response and immunogenic cell death as reported with TACE, strengthening the rationale for its use in combination with immunotherapy.^133^ The high dose of radiation is associated with necroptosis, *i.e.* caspase-independent apoptosis, which releases constitutive damage-associated molecular patterns resulting in immunogenic cell death.^134^ The initial immune activation is usually followed by local immunosuppressive action through recruitment of immune-suppressive subsets and enhanced expression of PD-L1 molecules.^26^ PD-1 and PD-L1 blockade by immunotherapy can overcome this immunosuppressive action and could be a potent synergistic treatment. The combination of radiation with immunotherapy in preclinical studies showed immune activation of the microenvironment and positive effects with the combination.^135^^,^^136^ Phase I/II trials testing the efficacy of SBRT with immunotherapy are underway (NCT03482102, NCT03203304, NCT03316872, NCT03817736).

## Conclusion

SBRT in HCC is safe and effective across the various BCLC stages whenever appropriately selected. Despite the lack of randomised phase III trials proving its efficacy, it appears to be an effective treatment alternative to RFA/TACE and is associated with long-term local control in most treated patients. Future trials are expected to bring it to the forefront but have been slow to accrue. Technical advances with proton and MRI-guided radiotherapy and combinations with immunotherapy are promising.

## Financial support

The authors received no financial support to produce this manuscript.

## Authors’ contributions

Study concept, design, acquisition of data: Shirley Lewis, Ali Hosni. Analysis and interpretation of data: All authors. Drafting of manuscript: All authors. Critical revision of manuscript for important intellectual content: All authors. Submission declaration: Not submitted or published previously.

## Conflict of interest

Shirley Lewis, Laura Dawson, Aisling Barry, Issa Mohamad- None. Teodor Stanescu- Research funding from Elekta for the development and implementation of novel imaging and treatment techniques for liver and pancreas on the Unity MR-Linac system. Ali Hosni- Nonfinancial leadership of liver TSG at Elekta MRL consortium.

Please refer to the accompanying ICMJE disclosure forms for further details.

## References

[1] Galle P.R., Forner A., Llovet J.M., Mazzaferro V., Piscaglia F., Raoul J.-L. (2018). EASL clinical practice guidelines: management of hepatocellular carcinoma. J Hepatol.

[2] Sung H., Ferlay J., Siegel R.L., Laversanne M., Soerjomataram I., Jemal A. (2021). Global cancer statistics 2020: GLOBOCAN estimates of incidence and mortality worldwide for 36 cancers in 185 countries. CA Cancer J Clin.

[3] Akinyemiju T., Abera S., Ahmed M., Alam N., Alemayohu M.A., Allen C. (2017). The burden of primary liver cancer and underlying etiologies from 1990 to 2015 at the global, regional, and national level: results from the global burden of disease study 2015. JAMA Oncol.

[4] Valery P.C., Laversanne M., Clark P.J., Petrick J.L., McGlynn K.A., Bray F. (2018). Projections of primary liver cancer to 2030 in 30 countries worldwide. Hepatology.

[5] Pons F., Varela M., Llovet J.M. (2005). Staging systems in hepatocellular carcinoma. HPB (Oxford).

[6] Marrero J.A., Kulik L.M., Sirlin C.B., Zhu A.X., Finn R.S., Abecassis M.M. (2018). Diagnosis, staging, and management of hepatocellular carcinoma: 2018 practice guidance by the American Association for the Study of Liver Diseases. Hepatology.

[7] Barry A., Apisarnthanarax S., O'Kane G.M., Sapisochin G., Beecroft R., Salem R. (2020). Management of primary hepatic malignancies during the COVID-19 pandemic: recommendations for risk mitigation from a multidisciplinary perspective. Lancet Gastroenterol Hepatol.

[8] Pinter M., Hucke F., Graziadei I., Vogel W., Maieron A., Königsberg R. (2012). Advanced-stage hepatocellular carcinoma: transarterial chemoembolization versus sorafenib. Radiology.

[9] Park S., Yoon W.S., Rim C.H. (2020). Indications of external radiotherapy for hepatocellular carcinoma from updated clinical guidelines: diverse global viewpoints. World J Gastroenterol.

[10] Serper M., Taddei T.H., Mehta R., D'Addeo K., Dai F., Aytaman A. (2017). Association of provider specialty and multidisciplinary care with hepatocellular carcinoma treatment and mortality. Gastroenterology.

[11] Herrero J.I., Sangro B., Pardo F., Quiroga J., Iñarrairaegui M., Rotellar F. (2008). Liver transplantation in patients with hepatocellular carcinoma across Milan criteria. Liver Transplant.

[12] Sempokuya T., Wong L.L. (2019). Ten-year survival and recurrence of hepatocellular cancer. Hepatoma Res.

[13] Singal A.G., Pillai A., Tiro J. (2014). Early detection, curative treatment, and survival rates for hepatocellular carcinoma surveillance in patients with cirrhosis: a meta-analysis. PLoS Med.

[14] Zhang B.-H., Yang B.-H., Tang Z.-Y. (2004). Randomized controlled trial of screening for hepatocellular carcinoma. J Cancer Res Clin Oncol.

[15] Stillwagon G.B., Order S.E., Guse C., Klein J.L., Leichner P.K., Leibel S.A. (1989). 194 hepatocellular cancers treated by radiation and chemotherapy combinations: toxicity and response: a Radiation Therapy Oncology Group Study. Int J Radiat Oncol Biol Phys.

[16] Rim C.H., Seong J. (2016). Application of radiotherapy for hepatocellular carcinoma in current clinical practice guidelines. Radiat Oncol J.

[17] Dawson L.A., Ten Haken R.K. (2005). Partial volume tolerance of the liver to radiation. Semin Radiat Oncol.

[18] Cheng S.H., Lin Y.M., Chuang V.P., Yang P.S., Cheng J.C., Huang A.T. (1999). A pilot study of three-dimensional conformal radiotherapy in unresectable hepatocellular carcinoma. J Gastroenterol Hepatol.

[19] Liu M.T., Li S.H., Chu T.C., Hsieh C.Y., Wang A.Y., Chang T.H. (2004). Three-dimensional conformal radiation therapy for unresectable hepatocellular carcinoma patients who had failed with or were unsuited for transcatheter arterial chemoembolization. Jpn J Clin Oncol.

[20] McIntosh A., Hagspiel K.D., Al-Osaimi A.M., Northup P., Caldwell S., Berg C. (2009). Accelerated treatment using intensity-modulated radiation therapy plus concurrent capecitabine for unresectable hepatocellular carcinoma. Cancer.

[21] Kong M., Hong S.E., Choi W.S., Choi J., Kim Y. (2013). Treatment outcomes of helical intensity-modulated radiotherapy for unresectable hepatocellular carcinoma. Gut Liver.

[22] Ben-Josef E., Normolle D., Ensminger W.D., Walker S., Tatro D., Ten Haken R.K. (2005). Phase II trial of high-dose conformal radiation therapy with concurrent hepatic artery floxuridine for unresectable intrahepatic malignancies. J Clin Oncol.

[23] Blomgren H., Lax I., Göranson H., Kræpelien T., Nilsson B., Näslund I. (1998). Radiosurgery for tumors in the body: clinical experience using a new method. J Radiosurgery.

[24] Brown J.M., Carlson D.J., Brenner D.J. (2014). The tumor radiobiology of SRS and SBRT: are more than the 5 Rs involved?. Int J Radiat Oncol Biol Phys.

[25] Park H.J., Griffin R.J., Hui S., Levitt S.H., Song C.W. (2012). Radiation-induced vascular damage in tumors: implications of vascular damage in ablative hypofractionated radiotherapy (SBRT and SRS). Radiat Res.

[26] Lee Y.H., Tai D., Yip C., Choo S.P., Chew V. (2020). Combinational immunotherapy for hepatocellular carcinoma: radiotherapy, immune checkpoint blockade and beyond. Front Immunol.

[27] Kang J.K., Kim M.S., Cho C.K., Yang K.M., Yoo H.J., Kim J.H. (2012). Stereotactic body radiation therapy for inoperable hepatocellular carcinoma as a local salvage treatment after incomplete transarterial chemoembolization. Cancer.

[28] Buckstein M., Kim E., Fischman A., Facciuto M., Schwartz M., Rosenzweig K. (2018). Stereotactic body radiation therapy following transarterial chemoembolization for unresectable hepatocellular carcinoma. J Gastrointest Oncol.

[29] Kibe Y., Takeda A., Tsurugai Y., Eriguchi T. (2020). Local control by salvage stereotactic body radiotherapy for recurrent/residual hepatocellular carcinoma after other local therapies. Acta Oncologica.

[30] Kim T.H., Koh Y.H., Kim B.H., Kim M.J., Lee J.H., Park B. (2021). Proton beam radiotherapy vs. radiofrequency ablation for recurrent hepatocellular carcinoma: a randomized phase III trial. J Hepatol.

[31] Yoon S.M., Kim S.Y., Lim Y.-S., Kim K.M., Shim J.H., Lee D. (2020). Stereotactic body radiation therapy for small (≤ 5 cm) hepatocellular carcinoma not amenable to curative treatment: results of a single-arm, phase II clinical trial. Clin Mol Hepatol.

[32] Durand-Labrunie J., Baumann A.-S., Ayav A., Laurent V., Boleslawski E., Cattan S. (2020). Curative irradiation treatment of hepatocellular carcinoma: a multicenter phase 2 trial. Int J Radiat Oncol Biol Phys.

[33] Jang W.I., Bae S.H., Kim M.S., Han C.J., Park S.C., Kim S.B. (2020). A phase 2 multicenter study of stereotactic body radiotherapy for hepatocellular carcinoma: safety and efficacy. Cancer.

[34] Park S., Jung J., Cho B., Kim S.Y., Yun S.-C., Lim Y.-S. (2020). Clinical outcomes of stereotactic body radiation therapy for small hepatocellular carcinoma. J Gastroenterol Hepatol.

[35] Mathew A.S., Atenafu E.G., Owen D., Maurino C., Brade A., Brierley J. (2020). Long term outcomes of stereotactic body radiation therapy for hepatocellular carcinoma without macrovascular invasion. Eur J Cancer.

[36] Kim J.W., Han K.-H., Seong J. (2019). Phase I/II trial of helical IMRT-based stereotactic body radiotherapy for hepatocellular carcinoma. Dig Liver Dis.

[37] Hara K., Takeda A., Tsurugai Y., Saigusa Y., Sanuki N., Eriguchi T. (2019). Radiotherapy for hepatocellular carcinoma results in comparable survival to radiofrequency ablation: a propensity score analysis. Hepatology.

[38] Feng M., Suresh K., Schipper M.J., Bazzi L., Ben-Josef E., Matuszak M.M. (2018). Individualized adaptive stereotactic body radiotherapy for liver tumors in patients at high risk for liver damage: a phase 2 clinical trial. JAMA Oncol.

[39] Takeda A., Sanuki N., Tsurugai Y., Iwabuchi S., Matsunaga K., Ebinuma H. (2016). Phase 2 study of stereotactic body radiotherapy and optional transarterial chemoembolization for solitary hepatocellular carcinoma not amenable to resection and radiofrequency ablation. Cancer.

[40] Lasley F.D., Mannina E.M., Johnson C.S., Perkins S.M., Althouse S., Maluccio M. (2015). Treatment variables related to liver toxicity in patients with hepatocellular carcinoma, Child-Pugh class A and B enrolled in a phase 1-2 trial of stereotactic body radiation therapy. Pract Radiat Oncol.

[41] Scorsetti M., Comito T., Cozzi L., Clerici E., Tozzi A., Franzese C. (2015). The challenge of inoperable hepatocellular carcinoma (HCC): results of a single-institutional experience on stereotactic body radiation therapy (SBRT). J Cancer Res Clin Oncol.

[42] Bujold A., Massey C.A., Kim J.J., Brierley J., Cho C., Wong R.K. (2013). Sequential phase I and II trials of stereotactic body radiotherapy for locally advanced hepatocellular carcinoma. J Clin Oncol.

[43] Andolino D.L., Johnson C.S., Maluccio M., Kwo P., Tector A.J., Zook J. (2011). Stereotactic body radiotherapy for primary hepatocellular carcinoma. Int J Radiat Oncol Biol Phys.

[44] Ackley B.J.L.G., Swan B.A., Tucker S.J. (2007).

[45] Yoon S.M., Ryoo B.Y., Lee S.J., Kim J.H., Shin J.H., An J.H. (2018). Efficacy and safety of transarterial chemoembolization plus external beam radiotherapy vs sorafenib in hepatocellular carcinoma with macroscopic vascular invasion: a randomized clinical trial. JAMA Oncol.

[46] Wei X., Jiang Y., Zhang X., Feng S., Zhou B., Ye X. (2019). Neoadjuvant three-dimensional conformal radiotherapy for resectable hepatocellular carcinoma with portal vein tumor thrombus: a randomized, open-label, multicenter controlled study. J Clin Oncol.

[47] Reig M., Forner A., Rimola J., Ferrer-Fábrega J., Burrel M., Garcia-Criado Á. (2022). BCLC strategy for prognosis prediction and treatment recommendation: the 2022 update. J Hepatol.

[48] Hallemeier C.L., Apisarnthanarax S., Dawson L.A. (2022). BCLC 2022 update: important advances, but missing external beam radiotherapy. J Hepatol.

[49] Apisarnthanarax S., Barry A., Cao M., Czito B., DeMatteo R., Drinane M. (2022). External beam radiation therapy for primary liver cancers: an ASTRO clinical practice guideline. Pract Radiat Oncol.

[50] Heimbach J.K., Kulik L.M., Finn R.S., Sirlin C.B., Abecassis M.M., Roberts L.R. (2018). AASLD guidelines for the treatment of hepatocellular carcinoma. Hepatology.

[51] Kulik L., Heimbach J.K., Zaiem F., Almasri J., Prokop L.J., Wang Z. (2018). Therapies for patients with hepatocellular carcinoma awaiting liver transplantation: a systematic review and meta-analysis. Hepatology.

[52] Bush D.A., Hillebrand D.J., Slater J.M., Slater J.D. (2004). High-dose proton beam radiotherapy of hepatocellular carcinoma: preliminary results of a phase II trial. Gastroenterology.

[53] Katz A.W., Chawla S., Qu Z., Kashyap R., Milano M.T., Hezel A.F. (2012). Stereotactic hypofractionated radiation therapy as a bridge to transplantation for hepatocellular carcinoma: clinical outcome and pathologic correlation. Int J Radiat Oncol Biol Phys.

[54] Sandroussi C., Dawson L.A., Lee M., Guindi M., Fischer S., Ghanekar A. (2010). Radiotherapy as a bridge to liver transplantation for hepatocellular carcinoma. Transpl Int.

[55] O'Connor J.K., Trotter J., Davis G.L., Dempster J., Klintmalm G.B., Goldstein R.M. (2012). Long-term outcomes of stereotactic body radiation therapy in the treatment of hepatocellular cancer as a bridge to transplantation. Liver Transplant.

[56] Mohamed M., Katz A.W., Tejani M.A., Sharma A.K., Kashyap R., Noel M.S. (2016). Comparison of outcomes between SBRT, yttrium-90 radioembolization, transarterial chemoembolization, and radiofrequency ablation as bridge to transplant for hepatocellular carcinoma. Adv Radiat Oncol.

[57] Nugent F.W., Qamar A., Stuart K.E., Galuski K., Flacke S., Molgaard C. (2017). A randomized phase II study of individualized stereotactic body radiation therapy (SBRT) versus transarterial chemoembolization (TACE) with DEBDOX beads as a bridge to transplant in hepatocellular carcinoma (HCC). J Clin Oncol.

[58] Nugent F.W., Hunter K., Molgaard C., Qamar A., Gunturu K., Stuart K.E. (2020). A randomized phase II feasibility study of individualized stereotactic body radiation therapy (SBRT) versus transarterial chemoembolization (TACE) with DEBDOX beads as a bridge to transplant in hepatocellular carcinoma (HCC). J Clin Oncol.

[59] Bush D.A., Smith J.C., Slater J.D., Volk M.L., Reeves M.E., Cheng J. (2016). Randomized clinical trial comparing proton beam radiation therapy with transarterial chemoembolization for hepatocellular carcinoma: results of an interim analysis. Int J Radiat Oncol Biol Phys.

[60] Sapisochin G., Barry A., Doherty M., Fischer S., Goldaracena N., Rosales R. (2017). Stereotactic body radiotherapy vs. TACE or RFA as a bridge to transplant in patients with hepatocellular carcinoma. An intention-to-treat analysis. J Hepatol.

[61] Chen M.-S., Li J.-Q., Zheng Y., Guo R.-P., Liang H.-H., Zhang Y.-Q. (2006). A prospective randomized trial comparing percutaneous local ablative therapy and partial hepatectomy for small hepatocellular carcinoma. Ann Surg.

[62] Waki K., Aikata H., Katamura Y., Kawaoka T., Takaki S., Hiramatsu A. (2010). Percutaneous radiofrequency ablation as first-line treatment for small hepatocellular carcinoma: results and prognostic factors on long-term follow up. J Gastroenterol Hepatol.

[63] Mathew A.S., Dawson L.A. (2021). Current understanding of ablative radiation therapy in hepatocellular carcinoma. J Hepatocell Carcinoma.

[64] Parikh N.D., Marshall V.D., Green M., Lawrence T.S., Razumilava N., Owen D. (2018). Effectiveness and cost of radiofrequency ablation and stereotactic body radiotherapy for treatment of early-stage hepatocellular carcinoma: an analysis of SEER-Medicare. J Med Imaging Radiat Oncol.

[65] Wahl D.R., Stenmark M.H., Tao Y., Pollom E.L., Caoili E.M., Lawrence T.S. (2016). Outcomes after stereotactic body radiotherapy or radiofrequency ablation for hepatocellular carcinoma. J Clin Oncol.

[66] Rajyaguru D.J., Borgert A.J., Smith A.L., Thomes R.M., Conway P.D., Halfdanarson T.R. (2018). Radiofrequency ablation versus stereotactic body radiotherapy for localized hepatocellular carcinoma in nonsurgically managed patients: analysis of the national cancer database. J Clin Oncol.

[67] Su T.S., Liang P., Zhou Y., Huang Y., Cheng T., Qu S. (2020). Stereotactic body radiation therapy vs. transarterial chemoembolization in inoperable barcelona clinic liver cancer stage a hepatocellular carcinoma: a retrospective, propensity-matched analysis. Front Oncol.

[68] Pan Y.-X., Fu Y.-Z., Hu D.-D., Long Q., Wang J.-C., Xi M. (2020). Stereotactic body radiotherapy vs. radiofrequency ablation in the treatment of hepatocellular carcinoma: a meta-analysis. Front Oncol.

[69] Lee J., Shin I.-S., Yoon W.S., Koom W.S., Rim C.H. (2020). Comparisons between radiofrequency ablation and stereotactic body radiotherapy for liver malignancies: meta-analyses and a systematic review. Radiother Oncol.

[70] Wang L., Ke Q., Huang Q., Shao L., Chen J., Wu J. (2020). Stereotactic body radiotherapy versus radiofrequency ablation for hepatocellular carcinoma: a systematic review and meta-analysis. Int J Hyperthermia.

[71] Culleton S., Jiang H., Haddad C.R., Kim J., Brierley J., Brade A. (2014). Outcomes following definitive stereotactic body radiotherapy for patients with Child-Pugh B or C hepatocellular carcinoma. Radiother Oncol.

[72] Ohri N., Tomé W.A., Méndez Romero A., Miften M., Ten Haken R.K., Dawson L.A. (2021). Local control after stereotactic body radiation therapy for liver tumors. Int J Radiat Oncol Biol Phys.

[73] Su T.-S., Liu Q.-H., Zhu X.-F., Liang P., Liang S.-X., Lai L. (2021). Optimal stereotactic body radiotherapy dosage for hepatocellular carcinoma: a multicenter study. Radiat Oncol.

[74] Llovet J.M., Bruix J. (2003). Systematic review of randomized trials for unresectable hepatocellular carcinoma: chemoembolization improves survival. Hepatology.

[75] Sapir E., Tao Y., Schipper M.J., Bazzi L., Novelli P.M., Devlin P. (2018). Stereotactic body radiation therapy as an alternative to transarterial chemoembolization for hepatocellular carcinoma. Int J Radiat Oncol Biol Phys.

[76] Bettinger D., Gkika E., Schultheiss M., Glaser N., Lange S., Maruschke L. (2018). Comparison of local tumor control in patients with HCC treated with SBRT or TACE: a propensity score analysis. BMC Cancer.

[77] Su T.-S., Lu H.-Z., Cheng T., Zhou Y., Huang Y., Gao Y.-C. (2016). Long-term survival analysis in combined transarterial embolization and stereotactic body radiation therapy versus stereotactic body radiation monotherapy for unresectable hepatocellular carcinoma> 5 cm. BMC cancer.

[78] Zhao J., Zeng L., Wu Q., Wang L., Lei J., Luo H. (2019). Stereotactic body radiotherapy combined with transcatheter arterial chemoembolization versus stereotactic body radiotherapy alone as the first-line treatment for unresectable hepatocellular carcinoma: a meta-analysis and systematic review. Chemotherapy.

[79] Munoz-Schuffenegger P., Barry A., Atenafu E.G., Kim J., Brierley J., Ringash J. (2021). Stereotactic body radiation therapy for hepatocellular carcinoma with Macrovascular invasion. Radiother Oncol.

[80] Bettinger D., Pinato D.J., Schultheiss M., Sharma R., Rimassa L., Pressiani T. (2019). Stereotactic body radiation therapy as an alternative treatment for patients with hepatocellular carcinoma compared to sorafenib: a propensity score analysis. Liver Cancer.

[81] Leibel S.A., Pajak T.F., Massullo V., Order S.E., Komaki R.U., Chang C.H. (1987). A comparison of misonidazole sensitized radiation therapy to radiation therapy alone for the palliation of hepatic metastases: results of a radiation therapy oncology group randomized prospective trial. Int J Radiat Oncology∗Biology∗Physics.

[82] Borgelt B., Gelber R., Brady L., Griffin T., Hendrickson F. (1981). The palliation of hepatic metastases: results of the Radiation Therapy Oncology Group pilot study. Int J Radiat Oncol Biol Phys.

[83] Soliman H., Ringash J., Jiang H., Singh K., Kim J., Dinniwell R. (2013). Phase II trial of palliative radiotherapy for hepatocellular carcinoma and liver metastases. J Clin Oncol.

[84] Koay E.J., Owen D., Das P. (2018). Radiation-induced liver disease and modern radiotherapy. Semin Radiat Oncol.

[85] Velec M., Haddad C.R., Craig T., Wang L., Lindsay P., Brierley J. (2017). Predictors of liver toxicity following stereotactic body radiation therapy for hepatocellular carcinoma. Int J Radiat Oncol Biol Phys.

[86] Sun J., Zhang T., Wang J., Li W., Zhang A., He W. (2019). Biologically effective dose (BED) of stereotactic body radiation therapy (SBRT) was an important factor of therapeutic efficacy in patients with hepatocellular carcinoma (≤5 cm). BMC Cancer.

[87] Toesca D.A., Osmundson E.C., Eyben R.V., Shaffer J.L., Lu P., Koong A.C. (2017). Central liver toxicity after SBRT: an expanded analysis and predictive nomogram. Radiother Oncol.

[88] Eriguchi T., Takeda A., Sanuki N., Oku Y., Aoki Y., Shigematsu N. (2013). Acceptable toxicity after stereotactic body radiation therapy for liver tumors adjacent to the central biliary system. Int J Radiat Oncol Biol Phys.

[89] Andolino D.L., Forquer J.A., Henderson M.A., Barriger R.B., Shapiro R.H., Brabham J.G. (2011). Chest wall toxicity after stereotactic body radiotherapy for malignant lesions of the lung and liver. Int J Radiat Oncol Biol Phys.

[90] Witt J.S., Rosenberg S.A., Bassetti M.F. (2020). MRI-guided adaptive radiotherapy for liver tumours: visualising the future. Lancet Oncol.

[91] Rosenberg S.A., Henke L.E., Shaverdian N., Mittauer K., Wojcieszynski A.P., Hullett C.R. (2019). A multi-institutional experience of MR-guided liver stereotactic body radiation therapy. Adv Radiat Oncol.

[92] Feldman A.M., Modh A., Glide-Hurst C., Chetty I.J., Movsas B. (2019). Real-time magnetic resonance-guided liver stereotactic body radiation therapy: an institutional report using a magnetic resonance-linac system. Cureus.

[93] Boldrini L., Romano A., Mariani S., Cusumano D., Catucci F., Placidi L. (2021). MRI-guided stereotactic radiation therapy for hepatocellular carcinoma: a feasible and safe innovative treatment approach. J Cancer Res Clin Oncol.

[94] Gaya A., Camilleri P., Nash A., Hughes D., Good J. (2021). Implementation of stereotactic MRI-guided adaptive radiotherapy (SMART) for hepatobiliary and pancreatic cancers in the United Kingdom - fifty in five. Cureus.

[95] Henke L., Kashani R., Robinson C., Curcuru A., DeWees T., Bradley J. (2018). Phase I trial of stereotactic MR-guided online adaptive radiation therapy (SMART) for the treatment of oligometastatic or unresectable primary malignancies of the abdomen. Radiother Oncol.

[96] Yoo G.S., Yu J.I., Park H.C. (2018). Proton therapy for hepatocellular carcinoma: current knowledges and future perspectives. World J Gastroenterol.

[97] Mizumoto M., Okumura T., Hashimoto T., Fukuda K., Oshiro Y., Fukumitsu N. (2011). Proton beam therapy for hepatocellular carcinoma: a comparison of three treatment protocols. Int J Radiat Oncol Biol Phys.

[98] Bush D.A., Kayali Z., Grove R., Slater J.D. (2011). The safety and efficacy of high-dose proton beam radiotherapy for hepatocellular carcinoma: a phase 2 prospective trial. Cancer.

[99] Fukuda K., Okumura T., Abei M., Fukumitsu N., Ishige K., Mizumoto M. (2017). Long-term outcomes of proton beam therapy in patients with previously untreated hepatocellular carcinoma. Cancer Sci.

[100] Hong T.S., Wo J.Y., Yeap B.Y., Ben-Josef E., McDonnell E.I., Blaszkowsky L.S. (2016). Multi-institutional phase II study of high-dose hypofractionated proton beam therapy in patients with localized, unresectable hepatocellular carcinoma and intrahepatic cholangiocarcinoma. J Clin Oncol.

[101] Kim T.H., Park J.-W., Kim B.H., Oh E.S., Youn S.H., Moon S.H. (2020). Phase II study of hypofractionated proton beam therapy for hepatocellular carcinoma. Front Oncol.

[102] Sanford N.N., Pursley J., Noe B., Yeap B.Y., Goyal L., Clark J.W. (2019). Protons versus photons for unresectable hepatocellular carcinoma: liver decompensation and overall survival. Int J Radiat Oncol Biol Phys.

[103] Bhangoo R.S., Mullikin T.C., Ashman J.B., Cheng T.W., Golafshar M.A., DeWees T.A. (2021). Intensity modulated proton therapy for hepatocellular carcinoma: initial clinical experience. Adv Radiat Oncol.

[104] Lazarev S., Hardy-Abeloos C., Factor O., Rosenzweig K., Buckstein M. (2018). Stereotactic body radiation therapy for centrally located hepatocellular carcinoma: outcomes and toxicities. J Cancer Res Clin Oncol.

[105] Herfarth K.K., Debus J., Lohr F., Bahner M.L., Rhein B., Fritz P. (2001). Stereotactic single-dose radiation therapy of liver tumors: results of a phase I/II trial. J Clin Oncol.

[106] Goodman K.A., Wiegner E.A., Maturen K.E., Zhang Z., Mo Q., Yang G. (2010). Dose-escalation study of single-fraction stereotactic body radiotherapy for liver malignancies. Int J Radiat Oncol Biol Phys.

[107] Meyer J.J., Foster R.D., Lev-Cohain N., Yokoo T., Dong Y., Schwarz R.E. (2016). A phase I dose-escalation trial of single-fraction stereotactic radiation therapy for liver metastases. Ann Surg Oncol.

[108] Jonsson J., Nyholm T., Söderkvist K. (2019). The rationale for MR-only treatment planning for external radiotherapy. Clin Transl Radiat Oncol.

[109] Tyagi N., Zelefsky M.J., Wibmer A., Zakian K., Burleson S., Happersett L. (2020). Clinical experience and workflow challenges with magnetic resonance-only radiation therapy simulation and planning for prostate cancer. Phys Imaging Radiat Oncol.

[110] Persson E., Jamtheim Gustafsson C., Ambolt P., Engelholm S., Ceberg S., Bäck S. (2020). MR-PROTECT: Clinical feasibility of a prostate MRI-only radiotherapy treatment workflow and investigation of acceptance criteria. Radiat Oncol.

[111] Fu J., Singhrao K., Cao M., Yu V., Santhanam A.P., Yang Y. (2020). Generation of abdominal synthetic CTs from 0.35T MR images using generative adversarial networks for MR-only liver radiotherapy. Biomed Phys Eng Express.

[112] Liu Y., Lei Y., Wang T., Kayode O., Tian S., Liu T. (2019). MRI-based treatment planning for liver stereotactic body radiotherapy: validation of a deep learning-based synthetic CT generation method. Br J Radiol.

[113] Zhang Q., Lou Y., Bai X.L., Liang T.B. (2020). Intratumoral heterogeneity of hepatocellular carcinoma: from single-cell to population-based studies. World J Gastroenterol.

[114] Barcena-Varela M., Lujambio A. (2021). The endless sources of hepatocellular carcinoma heterogeneity. Cancers (Basel).

[115] Gomes A.R., Abrantes A.M., Brito A.F., Laranjo M., Casalta-Lopes J.E., Gonçalves A.C. (2015). Influence of P53 on the radiotherapy response of hepatocellular carcinoma. Clin Mol Hepatol.

[116] Liu Y., Yan J., Wang F. (2018). Effects of TACE combined with precise RT on p53 gene expression and prognosis of HCC patients. Oncol Lett.

[117] Piao L.S., Hur W., Kim T.K., Hong S.W., Kim S.W., Choi J.E. (2012). CD133+ liver cancer stem cells modulate radioresistance in human hepatocellular carcinoma. Cancer Lett.

[118] Huang Y., Sheng H., Xiao Y., Hu W., Zhang Z., Chen Y. (2021). Wnt/β-catenin inhibitor ICG-001 enhances the antitumor efficacy of radiotherapy by increasing radiation-induced DNA damage and improving tumor immune microenvironment in hepatocellular carcinoma. Radiother Oncol.

[119] Ng S.S.W., Zhang H., Wang L., Citrin D., Dawson L.A. (2020). Association of pro-inflammatory soluble cytokine receptors early during hepatocellular carcinoma stereotactic radiotherapy with liver toxicity. NPJ Precis Oncol.

[120] Yamada I., Aung W., Himeno Y., Nakagawa T., Shibuya H. (1999). Diffusion coefficients in abdominal organs and hepatic lesions: evaluation with intravoxel incoherent motion echo-planar MR imaging. Radiology.

[121] Chen B.B., Shih T.T. (2014). DCE-MRI in hepatocellular carcinoma-clinical and therapeutic image biomarker. World J Gastroenterol.

[122] Zhou Y., Yang G., Gong X.-Q., Tao Y.-Y., Wang R., Zheng J. (2021). A study of the correlations between IVIM-DWI parameters and the histologic differentiation of hepatocellular carcinoma. Scientific Rep.

[123] Zheng J., Gong X.Q., Tao Y.Y., Wang R., Yang G., Li J.D. (2021). A correlative study between IVIM-DWI parameters and the expression levels of Ang-2 and TKT in hepatocellular carcinoma. Front Oncol.

[124] Shirota N., Saito K., Sugimoto K., Takara K., Moriyasu F., Tokuuye K. (2016). Intravoxel incoherent motion MRI as a biomarker of sorafenib treatment for advanced hepatocellular carcinoma: a pilot study. Cancer Imaging.

[125] Wu L., Xu P., Rao S., Yang L., Chen C., Liu H. (2017). ADC(total) ratio and D ratio derived from intravoxel incoherent motion early after TACE are independent predictors for survival in hepatocellular carcinoma. J Magn Reson Imaging.

[126] Chen B.B., Shao Y.Y., Lin Z.Z., Hsu C.H., Cheng A.L., Hsu C. (2021). Dynamic contrast-enhanced and intravoxel incoherent motion MRI biomarkers are correlated to survival outcome in advanced hepatocellular carcinoma. Diagnostics (Basel).

[127] Lambin P., Rios-Velazquez E., Leijenaar R., Carvalho S., van Stiphout R.G., Granton P. (2012). Radiomics: extracting more information from medical images using advanced feature analysis. Eur J Cancer.

[128] Wu K., Shui Y., Sun W., Lin S., Pang H. (2020). Utility of radiomics for predicting patient survival in hepatocellular carcinoma with portal vein tumor thrombosis treated with stereotactic body radiotherapy. Front Oncol.

[129] Harding-Theobald E., Louissaint J., Maraj B., Cuaresma E., Townsend W., Mendiratta-Lala M. (2021). Systematic review: radiomics for the diagnosis and prognosis of hepatocellular carcinoma. Aliment Pharmacol Ther.

[130] Dreher C., Linde P., Boda-Heggemann J., Baessler B. (2020). Radiomics for liver tumours. Strahlenther Onkol.

[131] Dogan N., Asher D., Farnia B., Ford C., Yang F., Portelance L. (2019). EP-2023 Predictive value of delta-radiomics features extracted from MR Images in image-guided liver SBRT. Radiother Oncol.

[132] Finn R.S., Qin S., Ikeda M., Galle P.R., Ducreux M., Kim T.-Y. (2020). Atezolizumab plus bevacizumab in unresectable hepatocellular carcinoma. New Engl J Med.

[133] Pinato D.J., Murray S.M., Forner A., Kaneko T., Fessas P., Toniutto P. (2021). Trans-arterial chemoembolization as a loco-regional inducer of immunogenic cell death in hepatocellular carcinoma: implications for immunotherapy. J ImmunoTherapy Cancer.

[134] Zhu M., Yang M., Zhang J., Yin Y., Fan X., Zhang Y. (2021). Immunogenic cell death induction by ionizing radiation. Front Immunol.

[135] Kim K.J., Kim J.H., Lee S.J., Lee E.J., Shin E.C., Seong J. (2017). Radiation improves antitumor effect of immune checkpoint inhibitor in murine hepatocellular carcinoma model. Oncotarget.

[136] Friedman D., Baird J.R., Young K.H., Cottam B., Crittenden M.R., Friedman S. (2017). Programmed cell death-1 blockade enhances response to stereotactic radiation in an orthotopic murine model of hepatocellular carcinoma. Hepatol Res.

[137] Benedict S.H., Yenice K.M., Followill D., Galvin J.M., Hinson W., Kavanagh B. (2010). Stereotactic body radiation therapy: the report of AAPM Task Group 101. Med Phys.

[138] Miften M., Vinogradskiy Y., Moiseenko V., Grimm J., Yorke E., Jackson A. (2021). Radiation dose-volume effects for liver SBRT. Int J Radiat Oncol Biol Phys.

[139] Hanna G.G., Murray L., Patel R., Jain S., Aitken K.L., Franks K.N. (2018). UK consensus on normal tissue dose constraints for stereotactic radiotherapy. Clin Oncol (R Coll Radiol).

